# Monodomain Blue Phase Liquid Crystal Layers for Phase Modulation

**DOI:** 10.1038/srep44575

**Published:** 2017-03-10

**Authors:** E. Oton, E. Netter, T. Nakano, Y. D.-Katayama, F. Inoue

**Affiliations:** 1 Nikon and Essilor International Joint Research Center Co., Ltd, KSP R&D Build. C10F-1032 3-2-1, Sakado, Takatsu-ku, Kawasaki-shi, Kanagawa 213-0012, Japan; 2 Essilor International SA, 147 rue de Paris, 94220, Charenton-Le-Pont, France; 3 Nikon Corporation, Shinagawa Intercity Tower C, 2-15-3, Konan, Minato-ku, Tokyo 108-6290, Japan

## Abstract

Liquid crystal “Blue Phases” (BP) have evolved, in the last years, from a scientific curiosity to emerging materials for new photonic and display applications. They possess attractive features over standard nematic liquid crystals, like submillisecond switching times and polarization- independent optical response. However, BPs still present a number of technical issues that prevent their use in practical applications: their phases are only found in limited temperature ranges, thus requiring stabilization of the layers; stabilized BP layers are inhomogeneous and not uniformly oriented, which worsen the optical performance of the devices. It would be essential for practical uses to obtain perfectly aligned and oriented monodomain BP layers, where the alignment and orientation of the cubic lattice are organized in a single 3D structure. In this work we have obtained virtually perfect monodomain BP layers and used them in devices for polarization independent phase modulation. We demonstrate that, under applied voltage, well aligned and oriented layers generate smoother and higher values of the phase shift than inhomogeneous layers, while preserving polarization independency. All BP devices were successfully stabilized in BPI phase, maintaining the layer monodomain homogeneity at room temperature, covering the entire area of the devices with a unique BP phase.

Chiral nematic liquid crystals (LC), commonly known as cholesteric liquid crystals (CLC), show a number of distinctive optical properties compared to achiral LCs, like selective spectral reflection and huge rotatory power. CLCs may also show peculiar colorful specific phases known as Blue Phases (BP). In recent years, BPs have evolved from a scientific curiosity to emerging materials for new applications^1^,^2^,^3^,^4^,^5^. Three different BP phases – BPI, BPII and BPIII - may appear at different temperature ranges. The most relevant phases from the practical point of view are BPI and BPII. The structure of these two phases, is constituted by an intricate double twist cylinder structure^6^ that is arranged in distinctive cubic lattices^7^,^8^,^9^,^10^. This cubic structure can be considered as a highly ordered isotropic medium. The most attractive features of BPs are submillisecond response times and optical isotropic behavior (light propagation independent of its polarization)^11^,^12^. These characteristics make them perfect candidates for a number of photonic and display applications^13^,^14^.

In particular, BP is among the limited number of solutions existing for achieving polarization independent tunable phase modulation with liquid crystal based devices. Tunable phase modulation has many different applications, for example, active lenses, aberration compensation, and beam steerers. Yet BPs still suffer from a small accessible phase shift linked to their inherent Kerr effect^15^. Consequently, improving phase shift, while preserving polarization independency, is a key parameter to use them in practical phase shift modulation devices.

Furthermore, BP phases are still only found at not so broad temperature ranges^16^, thus requiring stabilization of the layers for any practical application^17^. The most common method to stabilize BP layers is polymer stabilization. Besides, it has been found that alignment of BP phases can be achieved by standard procedures of surface conditioning, like usual nematics^18^. However, a difference in alignment behavior was shown between BPI and BPII^19^,^20^, and polymer stabilization and alignment conditioning were found to disturb BP orientation, making it difficult to obtain perfectly homogeneous layers.

External electric fields produce an isotropic elongation of the cubic polymer stabilized BP lattice. It follows that manufacturing of well aligned and oriented monodomain BP layers is essential for practical applications, especially in phase modulation devices. Indeed, an ordered structure of the BP cubes implies a smooth electrical switch of the liquid crystal, with all BP cubes elongated in the same fashion. This produces a significant improvement in the electrooptical properties of the devices^21^,^22^.

In this context, “monodomain” means that both, the alignment of the BP layer and the orientation of the cubic lattice are organized in a unique 3D structure over the whole layer. Homogeneous BPI layers have been previously reported in the literature, however they were multidomain layers^1^,^23^, not aligned^13^ and/or not stabilized at room temperature^22^,^24^.

In this work we have prepared Blue Phase layers with a virtually perfect monodomain structure and used them in devices for polarization independent phase modulation.

Furthermore, we demonstrate smoother and higher values of the phase shift with well aligned and oriented BP layers compared to not homogeneous layers, while preserving polarization independency under applied voltage. All manufactured Blue Phase devices were polymer stabilized in BPI phase; the monodomain homogeneity was maintained at room temperature, and the entire area of the devices was completely covered with a unique oriented BPI phase.

## Results and Discussion

### Blue phase monodomain layer analysis

Alignment layers play a key role in Blue Phase stabilization, as they change the temperature ranges where BPI and BPII show up. Moreover, alignment layers affect layer homogeneity as well as the orientation of the BP cubic structure. Blue Phase layer alignment and orientation were studied using rubbed polyimide as alignment layer in different cell thicknesses: the alignment and orientation were assessed by polarizing optical microscopy (POM) and Kossel pattern analysis^25^,^26^. Cells were manufactured and filled with the prepared liquid crystal mixture for Blue Phase, containing a guest liquid crystal LC-1 or LC-2. All cells underwent a temperature hysteresis analysis to find the appropriate temperature range where BPI and BPII appear. At that point, the alignment homogeneity was evaluated under the microscope by POM and then the phase and the orientation of the BP cubic structure were identified by Kossel pattern observation at different locations of the cell.

**Figure 1:**
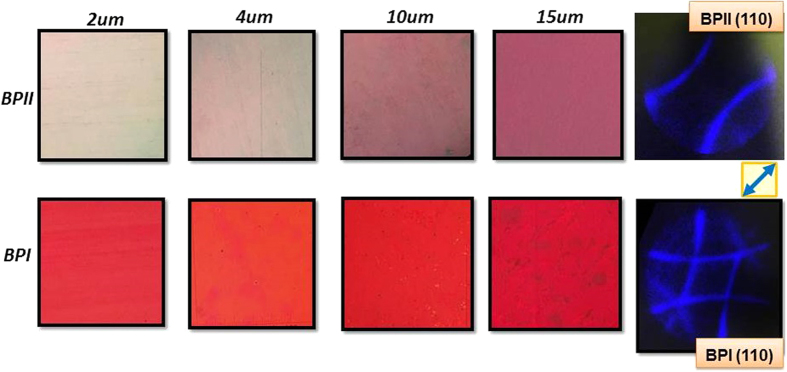
Micrographs of BPII (top) and BPI (bottom) aligned layers for different cell thicknesses obtained during the temperature hysteresis process and the corresponding Kossel pattern (the area of textures photos is 25 mm^2^ approx). The rubbing direction is indicated by the blue arrow.

As seen in Fig. 1, extremely well aligned homogeneous BPI and BPII layers were obtained for the whole batch from 2 to 15 μm. The textures of the BP layers remained constant and very uniform during the whole temperature range in which the phase was exhibited. The corresponding Kossel pattern of the layers is shown as well.

Figure 1 (top) corresponds to BPII layers, they showed a pale-reddish color that becomes darker as their thickness increases. A clear Kossel pattern (baseball pattern) that corresponds to the (110) cubic lattice orientation (simple cubic) was obtained. (110) orientation means that four parallel edges of the BP cube and none of the faces are parallel to the substrates. BPI layers, shown in the bottom row of Fig. 1, exhibited a bright red color and a characteristic (110) orientation (body-centered cubic).

The orientation of the Kossel pattern itself, for both BPII and BPI was 45° with respect of the rubbing direction; this orientation is maintained along the whole surface of the samples, *i.e*., there is a *unique* 3D monodomain BP structure with no platelets or otherwise oriented domains. Hence, the cubic structure of the Blue Phase is oriented at 45° with respect of the rubbing direction as well. This behavior can be explained with the observation of the BP layer transition as the sample is being cooled down from BPII to BPI.

**Figure 2:**
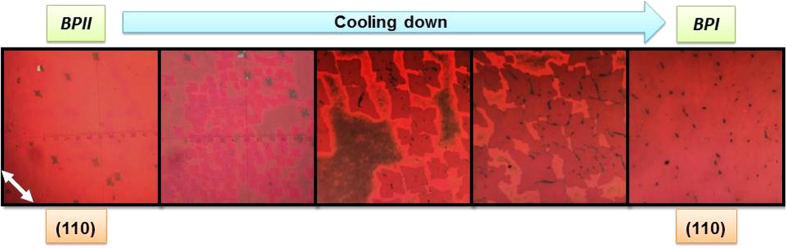
Micrographs of the transition between BPII and BPI when cooling down. Transition to BPI is characterized by the appearance of (110) crystals oriented at 45°. The rubbing direction is indicated by the white arrow.

When the BPII layer is cooled down at a slow rate, structures with a rhombic shape appear scattered over the surface and start growing (Fig. 2). These structures, when analyzed by Kossel pattern, reveal they consist of a BPI (110) orientation. This is the starting point of BPI phase. The crystalline shape of a single BPI crystal, as observed from the [110] direction, resembles a rhombic shape^25^, thus it is fair to assume this is directly related to the crystal habit, the crystalline shape of individual crystals growing. In our case, the preferential growing direction of the BPI crystals occurs at 45° (with respect of the rubbing direction) and this fact is supported by the Kossel pattern observation that is oriented at 45° as well.

Previous works have reported monodomain BPII structures^19^,^20^. However, to our knowledge, monodomain BPI structures have not been previously achieved. The difference of alignment behavior between BPI and BPII was attributed to a difference in the configuration of their disclination lines.

In BPII, the disclination lines of the cubic crystal are not separated^27^. Connected disclinations lines imply that a monodomain can be formed because it corresponds to a single global minimum of free energy. In BPI, where the disclination lines are separated, there are several local minima of free energy, so the cubic lattice orientation will tend to reach one of the closest minima, resulting, in general, in a multidomain BPI structure^1^. However, in previous works, BPI is generally obtained by a heating process from cholesteric phase, meaning that the free energy can go into any local minimum. In our case, however, BPI is obtained by cooling down from BPII. Thus, although the kinetics of the phase transition and change of structure between BPII and BPI has not yet been proved theoretically, our hypothesis is that, in the transition from BPII to BPI, the cubic lattice monodomain orientation can be maintained, because the energy minimum of BPII is very close to a local minimum of the resulting BPI. For this reason, the mismatch in the crystal orientation is very small, cubic lattice does not need to rotate and BPI remains in the same orientation as BPII.

In general, the predominant BP crystal orientation obtained in previous reported studies is (100). The orientation of the BP cubic lattice depends on a number of factors, a very influential one being the chiral dopant concentration. The cubic lattice orientation can change abruptly, when the concentration of the chiral dopant is over a certain value^28^. For this study, the chiral dopant that was used has a relatively high HTP, which can influence and change the lattice orientation even at lower concentrations.

Before polymer stabilization of BPI layers, a study with a Vis-UV spectrophotometer (in reflection mode) was performed to confirm the phase of the BP, the orientation of the cubic structure and the lattice size by means of a Kossel pattern generator.

The Kossel pattern generator aims to calculate, providing the Bragg reflection peak position and the measurement optical system characteristics are known, all the possible Kossel diffraction patterns, taking into account the crystalline structure (blue phase I or II), orientation, and size of the lattice. The computed patterns are then compared to the experimental patterns, allowing us to identify involved diffraction planes, orientation and lattice size.

**Figure 3:**
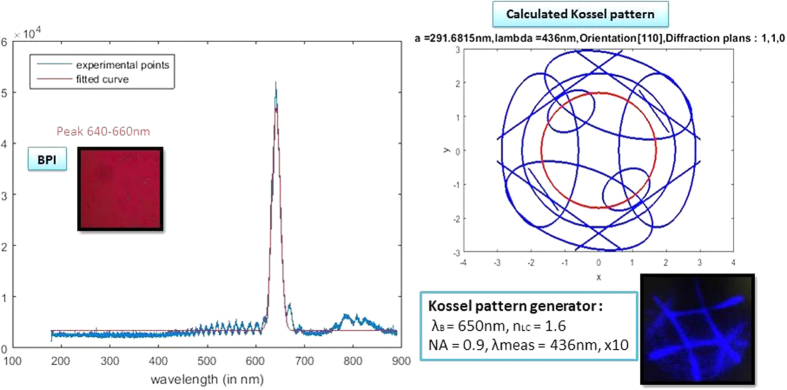
Reflection spectrum measured for the BPI layer with LC-1 mixture (left). The measured BPI layer is shown in the inset and shows a peak at 650 nm. The Kossel pattern generated from a 650 nm Bragg reflectance peak (top, right) corresponds to a BPI (110) and a calculated lattice size of 292 nm. The red circle is the field of view of the observed Kossel pattern (bottom, right).

The spectra were taken while decreasing the temperature from BPII to BPI. In the range where the Kossel observation would give a BPI (110) pattern, the spectrum peak was found to be around 650 nm in all cases (Fig. 3, left). There were minor variations due to small layer thickness variations. To confirm the BP phase and orientation, the input data were assumed to be a Bragg peak of 650 nm. The only plausible solution for this Bragg peak is having a (110) orientation, which gives the Kossel pattern experimentally observed (Fig. 3, right). For this Bragg peak and Kossel pattern, the calculated lattice size for BPI is 292 nm.

**Figure 4:**
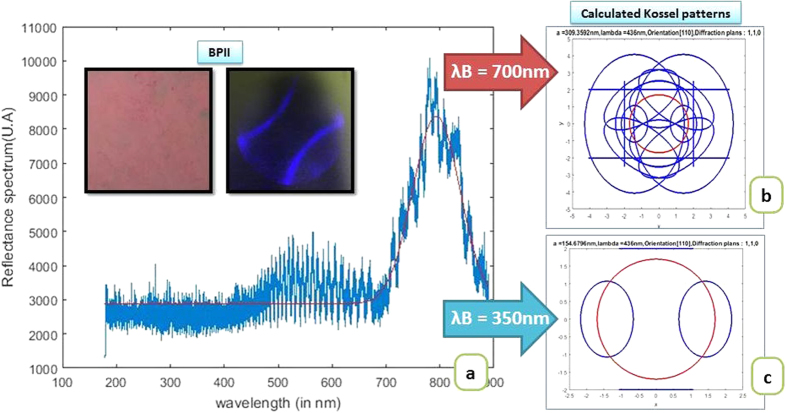
Reflection spectrum measured for the BPII layer with LC-1 mixture (**a**). The measured BPII layer and corresponding Kossel pattern are shown in the inset. The spectrum shows a peak at 700 nm. The Kossel patterns calculated for a 700 nm reflectance peak (**b**) and a 350 nm peak (**c**), which corresponds to the baseball pattern observed. The red circle is the field of view of the observed Kossel pattern. The calculated lattice size is 155 nm.

Equivalent calculations were done for BPII layers. A wide peak around 700 nm appeared in this temperature range that shifted towards smaller wavelengths when decreasing the temperature (Fig. 4(a)). When performing the calculation in the Kossel pattern generator, assuming the reflectance peak occurs at 700 nm, the generated Kossel patterns do not agree with the experimental observation. No pattern corresponds to the one found experimentally (Fig. 4(b)). It is known that BPII lattices are smaller than their BPI lattices size (their cubic structure is *sc* and *bcc* respectively)^25^, so the reflectance peak found at 700 nm might be due to noise from the halogen lamp instead. The reflectance peak might not be seen with the spectrophotometer because it is found in the UV area and the optical equipment cuts off this part of the spectrum. Working under these assumptions, the input data in the Kossel pattern generator was set to a value under the spectral cut. We assumed the reflectance peak to be at 350 nm. The calculated Kossel pattern that was obtained was, then, the typical baseball shaped pattern (Fig. 4(c)), that corresponds to BPII (110) and matched the experimental observation. The estimated lattice size for BPII is 155 nm.

The same temperature hysteresis process and Kossel analysis were performed for the Blue Phase with LC-2 guest mixture. The alignment, structure and orientation found in the LC-2 mixture samples are completely equivalent to the ones of LC-1. Well aligned homogeneous BPI layers were obtained with a uniform orientation at (110) that is oriented at 45° of the rubbing direction of the substrates. Spectrophotometry was employed to measure these samples as well, to confirm the phase, the orientation with the Kossel generator and the lattice size. The spectra obtained for the BP layers with LC-2 mixture were equivalent to those of LC-1: a peak in the range of 640–660 nm was found for BPI and the Kossel pattern confirmed the orientation (110).

### Polymerized Blue Phase monodomain layers

**Figure 5:**
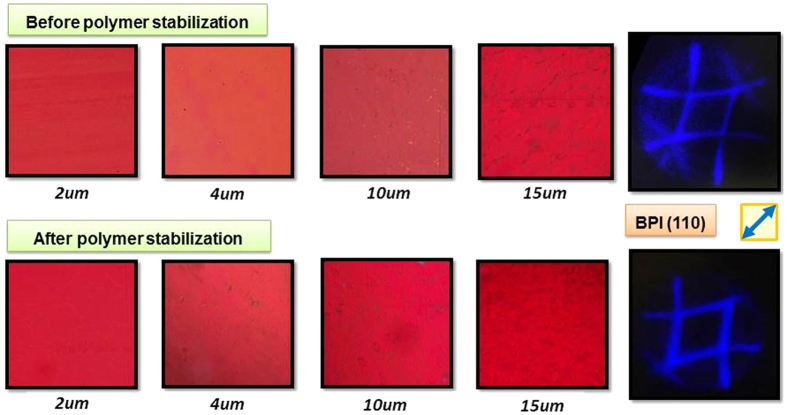
Micrographs of the BPI aligned layers with LC-1 mixture for different cell thicknesses and corresponding Kossel pattern: before and after polymer stabilization (top and bottom), (the area of texture photos is 25 mm^2^ approx). The rubbing direction is indicated by the blue arrow.

Figure 5 shows BPI aligned layers before and after the polymer stabilization process for LC-1, (equivalent layers were obtained for LC-2). The “after polymerization” photos were taken at room temperature. The layer homogeneity and texture were controlled during the polymer stabilization process. During the polymerization, the polymer chains are condensed in the disclination lines of the BP cubic structure that stabilize it^6^. By performing the polymerization process at an extremely slow rate, we were able to maintain the alignment and orientation of the BP layers undisturbed after finishing the process. The UV irradiance is *at least* one order of magnitude lower than previously described polymerization methods^1^,^17^,^18^.

The BPI alignment and the orientation of the cubic structure were checked after polymerization. For both LC-1 and LC-2 mixtures and for every cell gap, the Kossel pattern analysis at room temperature demonstrated that the orientation of the BPI did not change after the polymerization process, (Fig. 5). The orientation was maintained for BPI (110) with the BP cubes oriented at 45° to the rubbing direction on the whole surface of the devices.

The number of BP cubes squeezed between the substrates is a rather small integer (around 25 cubes in a 10 μm gap cell). Small layer thickness differences result in slightly lower or higher number of cubes. This is seen as darker areas or brighter areas since reflected power is slightly lower or higher. Nevertheless, these areas are not inhomogeneities, as the Kossel pattern does not change, indicating that the cubic structure and orientation is kept.

### Phase shift characterization

The phase shift study was done by Mach-Zehnder interferometry. The devices were measured at room temperature after the polymer stabilization process. Phase shift measurements were performed in cells with different thicknesses with non-coated and polyimide coated layers respectively for comparison purposes. Both LC mixtures were measured as well.

#### Non-coated surfaces vs. rubbed PI surfaces

When comparing non-coated and rubbed polyimide samples, evident differences are found between them, both on the BP behavior and the physical properties. Several factors were compared: saturation voltage (*Vsat*) and threshold voltage (*Vth*), maximum phase shift, and layer homogeneity and alignment. In general, saturation voltage *Vsat*, and threshold voltage *Vth*, occur at lower voltages for non-rubbed samples. This makes sense, as the cubic structure is not oriented or aligned within the surface, meaning the BP is less attached (the anchoring energy is lower), to the surfaces in the non-rubbed cells.

**Figure 6:**
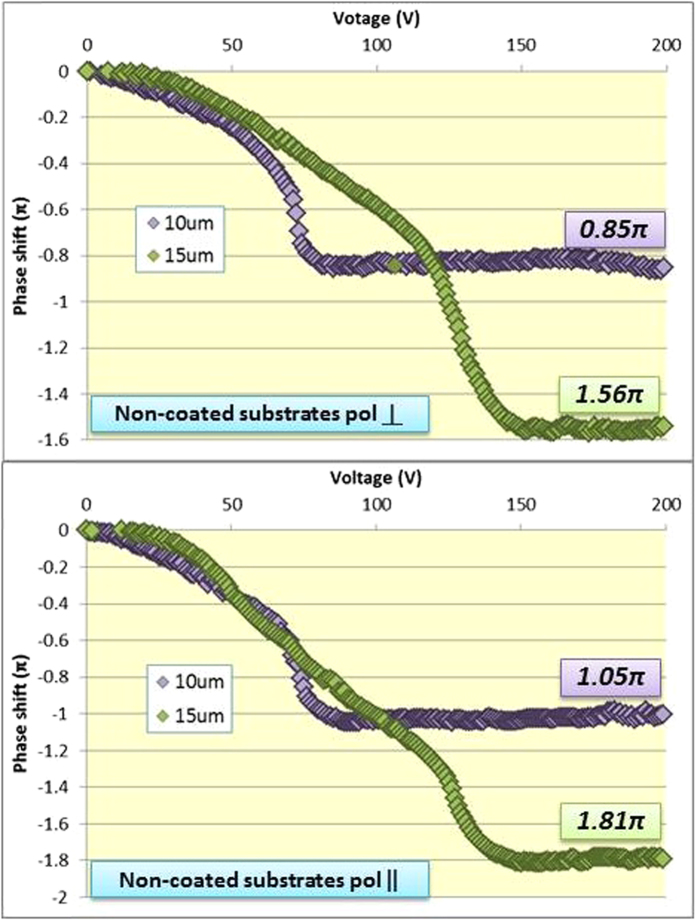
Phase shift measured for the Blue Phase with LC-1 mixture in non-coated cells for the two orthogonal polarizations.

As seen in Fig. 6, the maximum phase shift obtained for the non-coated cells with LC-1 mixture was 1.81π for the 15 μm thick samples and the parallel polarization. However, a double slope of the phase shift variation was observed. This is attributed to two different switching effects taking place: BP molecule reorientation can occur at lower applied voltages, allowing for a uniform variation of the phase shift. Then, if the voltage is further increased, the *electrostriction* effect can take place, where the BP lattice is actually distorted towards the applied field^25^.

Additionally, the maximum phase shift obtained for the two orthogonal polarizations for these cells was not the same, having a difference even of 0.2π in some cases. As previously explained the layer homogeneity obtained for non-coated surfaces was very low, therefore different BP cube deformations can occur when voltage is applied (in different axes), especially when electrostriction occurs: there is no polarization independency and that might explain why the two orthogonal polarization performances are different from each other.

The maximum phase shifts obtained at saturation voltages for BP layers in rubbed PI are higher than for non-coated surfaces: 1.4π and 1.05π respectively for 10 μm cells. The slopes of the phase shift curves vary evenly in the whole field region: well aligned and oriented blue phase layers allow for a smooth and ordered switching, so that the cubic structure is able to switch in the same manner. For this reason a higher contribution to the phase shift and better electrooptical performances can be obtained while polarization independency is preserved.

#### LC-1 and LC-2 mixtures phase shift in rubbed PI surfaces

A number of cells with LC-1 or LC-2 mixture and different thicknesses were measured and compared. As the birefringence for the LC-1 guest is lower than the one of LC-2, its Kerr constant is also lower. As a result, the phase shift coverage for this mixture is expected to be lower in LC-1 than in LC-2 for the same applied voltage because this quantity is proportional to the Kerr constant.

In this case *Vth* and *Vsat* for both types of samples are not significantly different at a fixed cell thickness. However the maximum applied voltage that our equipment could reach was 200 V which was not enough to achieve the saturation voltage in the thickest thicknesses.

**Figure 7:**
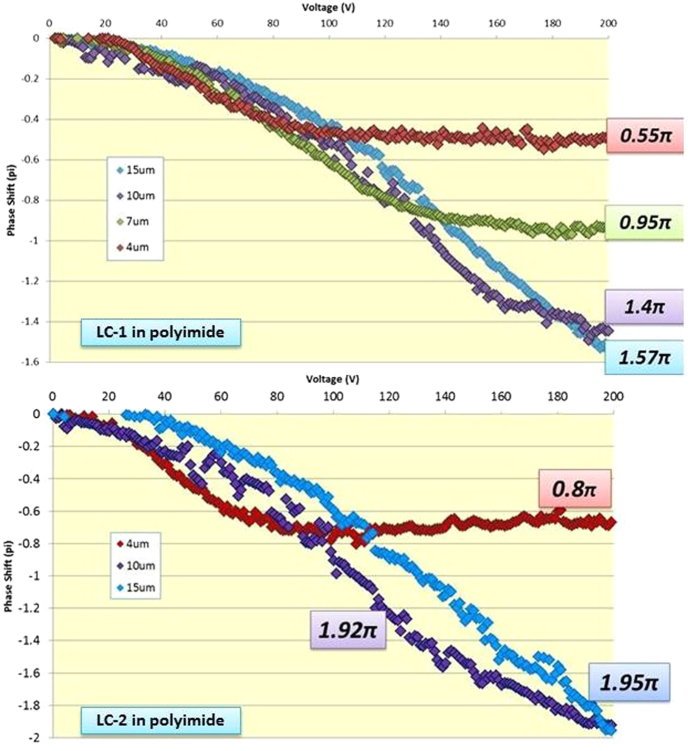
Phase shift measured for the Blue Phase with LC-1 and LC-2 mixtures in rubbed polyimide .

As seen in Fig. 7, the maximum phase shift achieved for the 7 μm, 10 μm and 15 μm cells were 0.95π, 1.4π and 1.57π respectively for LC-1. Note that the 15 μm curve crossed over the 10 μm, which is nearly saturated. From the measured response curve, it is clear that the 15 μm cells do not reach *Vsat* within the 200 V range. With extrapolated data from all the measured samples, a 1.7π phase shift, saturation is expected to be achieved at 225 V.

Using LC-2, the maximum achieved phase shifts were higher than those for LC-1, reaching 0.8π for the 4 μm cells and 1.92π for the 10 μm cells, (Fig. 7, bottom). Notice that the *Vsat* is just achieved at 200 V for the 10 μm cells, which means that a thicker cell would eventually need more applied voltage. Nevertheless, 15 μm cells were also measured, reaching a phase shift of 1.95π at 200 V. From the extrapolation of the measured cells data the 15 μm with LC-2 cells would produce 2.3π phase shift at 225 V.

Every phase shift measurement was performed for two orthogonal polarizations of the impinging laser light. The electrooptical response and maximum achieved phase shift values were essentially the same for both polarizations in all cases (with variations of 0.05π at most), thus demonstrating the polarization independency of the devices.

## Conclusions

Virtually perfectly organized BP layers were successfully stabilized in devices at room temperature. A polarization independent phase shift modulator based on well oriented monodomain and stabilized blue phase layers has been demonstrated. The Kossel pattern analysis was essential to find out the phase of the BP and the orientation of such phase for the alignment and orientation control before the polymer stabilization process. The alignment homogeneity and orientation of the layers were proven to be key parameters to obtain smoother electro-optical responses and higher values of the phase shift than of those that are not oriented, reaching a 2π phase shift at 532 nm.

## Methods

The preparation of monodomain blue phase layers was performed in several stages. First, a number of different alignment layers were tested with the LC mixtures, in order to find a suitable one that would both allow the exhibition of the blue phase in a reasonable temperature range and also align the blue phase in a controlled orientation. The blue phase exhibition was studied with the temperature hysteresis and the alignment and layer homogeneity by polarizing optical microscopy (POM).

The phase and the orientation of the blue phase cubic structure were assessed by means of Kossel pattern analysis. Indeed, blue phase is known to have a three-dimensional periodical structure such as it exhibits a body centered cubic symmetry in the case of BPI and a simple cubic symmetry in the case of BPII. Thus, as in any crystal, if some convergent light enters a blue phase structure, a part of incident light meeting the Bragg equation is reflected back and generates a specific pattern which depends on its lattice spacing *a* and lattice orientation [*hkl*]^26^. This specific diffraction pattern is called Kossel pattern. As the lattice size of blue phase cubic structure is several 100’s of nm, its Kossel pattern can be observed by optical microscopy.

A number of cells with different alignment layers were prepared: (1) Polyimide layers (Nissan Chemical Industries, Ltd. Japan): alignment layers used in standard LC device manufacturing processes, (2) Optool (Daikin): fluoro-coating surfactant layers having a reduced surface free energy, (3) ITO-coated glass: glass substrates used without any alignment layer.

All deposited layers were studied in two conditions: untreated and rubbed. The polyimide layer was already deposited on the substrates (from EHC Co Ltd). For the Optool surfactant, the layers were deposited on clean substrates (without any layer) by dip coating, and then half of them went also through the rubbing process. In case of the glass-ITO surface cells were assembled directly from the clean substrates. After assembly, all cells were filled up with the LC mixture in isotropic state: The LC mixture was composed of a liquid crystal mixture LC-1, (with CB-5 as a guest, (Δn_1_ = 0.19)) with nematogen mixture LC JC1041XX and chiral dopant ISO(6OBA)_2_. The analyzed cell thicknesses were: 2, 4, 7, 10 and 15 µm. The active area of the cell, *i.e*., the ITO coated glass area where the alignment layer is deposited, is 1 cm^2^.

The homogeneity and orientation of the BP layers was assessed by: (1) Liquid crystal alignment: texture analysis by polarizing optical microscopy, (2) Temperature hysteresis cycle: controlled heating and cooling of the samples to find the temperature ranges where the phases BPI and BPII appear, and (3) Kossel pattern analysis: obtained after any phase change to determine what type of phase is observed and the orientation of the BP cubes.

The prepared cells underwent temperature hysteresis cycles to find out the temperature range where the blue phases appear. Blue Phase in both phases BPI and BPII was found in all tested layers at different temperature ranges.

**Figure 8:**
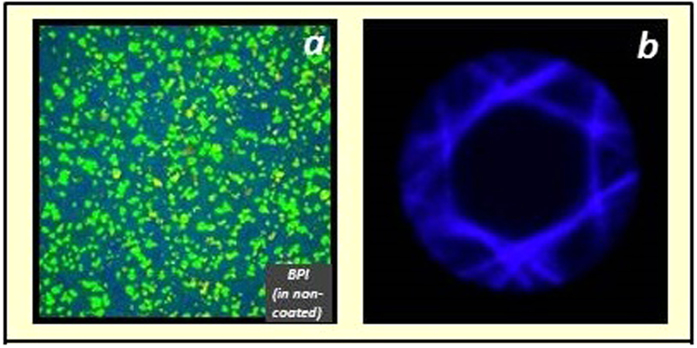
BPI platelets on uncoated substrates (**a**) and its Kossel pattern (**b**).

Figure 8a shows an example of BPI found in non-coated substrates. The Blue Phase layer exhibits a non-organized platelet texture. That means the Blue Phase layer is not homogeneous but the crystals are scattered all over the surface. This behavior occurred in all uncoated ITO-glass substrates, Optool layers (rubbed and non-rubbed), and non-rubbed polyimide layers. The consequence of a poor alignment is that a mixed or overlapped Kossel pattern is observed, and the reason is the different oriented BP cubes produce different diffraction planes (Fig. 8b). Thus, in scattered platelet structure, the cubic orientation of the Blue Phase is disorganized and shows no preferential orientation.

However, for rubbed polyimide layers, remarkably well aligned layers are obtained, as seen in Fig. 1. The exhibition of BPI, the texture of the layers is very homogeneous and platelets are no longer seen, and a very well defined Kossel pattern indicates the excellent homogeneity of the layers.

Rubbed polyimide was used for obtaining monodomain BP layers and prepared for polymer stabilization and phase modulation. Substrates were assembled into 2, 4, 7, 10 and 15 μm cells and filled in isotropic state with the LC mixture prepared with monomers: Two different LC mixtures were prepared where the guest LC was changed, LC-1 or LC-2: LC-1 = CB5 (4-Cyano-4′-pentylbiphenyl) (Altair Co) Δn_1_ = 0.19 and LC-2 = MLC-2140 (Merck) Δn_2_ = 0.25, with nematogenic compound LC JC-1041XX (JNC Co, Japan), and chiral dopant ISO(6OBA)_2_ (Midori Kagaku Co., Ltd). Then two monomers RM257 (Wako Pure Chemical Industries Ltd) and 12CA (Altair Co), and photoinitiator Irgacure 184 (Altair Co) were added to the LC mixtures. For LC-1, the observed phase sequence is: CLC/43.6 °C/BPI/44.7 °C/BPII/45.8 °C/ISO; and for LC-2, the phase sequence is: CLC/65.0 °C/BPI/67.1 °C/BPII/68.8 °C/ISO.

Kossel patterns are observed with a Nikon Eclipse LV100POL polarized microscope (conoscopic image) with reflecting lighting and Bertrand lens. Illumination system consists of a halogen lamp and a band-pass interference Δλ filter centered around λ_0_ = 436 nm. Optical Kossel pattern is obtained at the pupil plane of the X100 objective lens with a high numerical aperture (NA = 0.85/ 0.9), focal length f’ (200 μm/100) with compensation ring (Nikon CFI Plan LWD IMSI 100X NA0.85). The Kossel patterns shown in the Figures correspond to 10 μm thick BP layers. Other thicknesses showed equivalent patterns.

For the polymer stabilization process the cells underwent temperature hysteresis cycle to find out the BP temperature ranges and analyzed by Kossel pattern to determine the phase and orientation of the BP cubic structure. The polymerization temperature was fixed where BPI is present. The polymerization process was carried out at 44.2 °C and 66.0 °C for LC-1 and LC-2 respectively and the samples were irradiated with a built in-house UV light system at 0.2 mW/cm^2^ (365 nm) for 30 minutes.

Photographs of the BP layers were taken with a polarizing optical microscope Nikon Eclipse LV100POL, with a commercial hot stage Instec mK2000. Spectra were taken with Ocean Optics USB4000 spectrophotometer.

## Additional Information

**How to cite this article:** Oton, E. *et al*. Monodomain Blue Phase Liquid Crystal Layers for Phase Modulation. *Sci. Rep.* **7**, 44575; doi: 10.1038/srep44575 (2017).

**Publisher's note:** Springer Nature remains neutral with regard to jurisdictional claims in published maps and institutional affiliations.
